# Modification of the Gastric Mucosal Microbiota by a Strain-Specific Helicobacter pylori Oncoprotein and Carcinogenic Histologic Phenotype

**DOI:** 10.1128/mBio.00955-19

**Published:** 2019-05-28

**Authors:** Jennifer M. Noto, Joseph P. Zackular, Matthew G. Varga, Alberto Delgado, Judith Romero-Gallo, Matthew B. Scholz, M. Blanca Piazuelo, Eric P. Skaar, Richard M. Peek

**Affiliations:** a Division of Gastroenterology, Department of Medicine, Vanderbilt University Medical Center, Nashville, Tennessee, USA; b Department of Pathology and Laboratory Medicine, University of Pennsylvania, Philadelphia, Pennsylvania, USA; c Children’s Hospital of Philadelphia, Philadelphia, Pennsylvania, USA; d Department of Epidemiology, University of North Carolina Gillings School of Global Public Health, Chapel Hill, North Carolina, USA; e Lineberger Comprehensive Cancer Center, Chapel Hill, North Carolina, USA; f Vanderbilt Technologies for Advanced Genomics, Vanderbilt University Medical Center, Nashville, Tennessee, USA; g Department of Pathology, Microbiology and Immunology, Vanderbilt University Medical Center, Nashville, Tennessee, USA; New York University School of Medicine

**Keywords:** CagA, *Helicobacter pylori*, gastric cancer, gastric microbiota, iron deficiency

## Abstract

Helicobacter pylori is the strongest risk factor for gastric adenocarcinoma; however, most infected individuals never develop this malignancy. Strain-specific microbial factors, such as the oncoprotein CagA, as well as environmental conditions, such as iron deficiency, augment cancer risk. Importantly, dysbiosis of the gastric microbiota is also associated with gastric cancer. To investigate the combinatorial effects of these determinants in an *in vivo* model of gastric cancer, Mongolian gerbils were infected with the carcinogenic *cag^+^* H. pylori strain 7.13 or a 7.13 *cagA* isogenic mutant, and microbial DNA extracted from gastric tissue was analyzed by 16S rRNA sequencing. Infection with H. pylori significantly increased gastric inflammation and injury, decreased α-diversity, and altered microbial community structure in a *cagA-*dependent manner. The effect of iron deficiency on gastric microbial communities was also investigated within the context of infection. H. pylori-induced injury was augmented under conditions of iron deficiency, but despite differences in gastric pathology, there were no significant differences in α- or β-diversity, phyla, or operational taxonomic unit (OTU) abundance among infected gerbils maintained on iron-replete or iron-depleted diets. However, when microbial composition was stratified based solely on the severity of histologic injury, significant differences in α- and β-diversity were present among gerbils harboring premalignant or malignant lesions compared to gerbils with gastritis alone. This study demonstrates that H. pylori decreases gastric microbial diversity and community structure in a *cagA-*dependent manner and that as carcinogenesis progresses, there are corresponding alterations in community structure that parallel the severity of disease.

## Abstract (precis)

Microbial communities are essential for the maintenance of human health, and when these communities are altered, hosts can become susceptible to inflammation and disease. Dysbiosis contributes to gastrointestinal cancers, and specific bacterial species are associated with this phenotype. This study uses a robust and reproducible animal model to demonstrate that H. pylori infection induces gastric dysbiosis in a *cagA*-dependent manner and further that dysbiosis and altered microbial community structure parallel the severity of H. pylori-induced gastric injury. Ultimately, such models of H. pylori infection and cancer that can effectively evaluate multiple determinants simultaneously may yield effective strategies for manipulating the gastric microbiota to prevent the development of gastric cancer.

## INTRODUCTION

Gastric adenocarcinoma is the third leading cause of cancer-related death worldwide, resulting in more than 780,000 deaths annually (1). The strongest known risk factor for this disease is infection with the gastric pathogen Helicobacter pylori. However, despite worldwide colonization rates of greater than 50%, only 1 to 3% of H. pylori-infected individuals ever develop gastric cancer. Drivers of susceptibility to disease include H. pylori strain-specific virulence factors, environmental conditions, and host determinants. One microbial genetic element that significantly increases the risk for gastric cancer is the *cag* pathogenicity island, which encodes a bacterial type IV secretion system (T4SS) that translocates the effector protein CagA into host gastric epithelial cells. Transgenic mice that overexpress CagA develop gastric epithelial cell hyperproliferation and gastric adenocarcinoma (2), further implicating CagA as a bacterial oncoprotein. One environmental condition associated with increased gastric cancer risk is iron deficiency (3, 4), and we previously demonstrated that iron deficiency significantly augments H. pylori-induced inflammation and the development of gastric adenocarcinoma *in vivo* (5). In addition to these risk factors, the microbiota of the stomach may also influence carcinogenesis.

In the intestine, microbial communities are essential for the maintenance of human health (6), and when the communities are altered, hosts can become susceptible to invading pathogens, with consequential inflammation and carcinogenesis (7). Dysbiosis can drive the development of gastrointestinal cancers, and specific bacterial species contribute to this phenotype (8–14). In the stomach, advances in DNA sequencing and analytical methods have revealed a complex human gastric microbiota (15); thus, interactions between the microbial community of the stomach and H. pylori may affect gastric pathophysiology and modulate disease (16), but these relationships have yet to be thoroughly defined.

Mongolian gerbils are a useful model to study H. pylori infection and gastric carcinogenesis, as infection closely recapitulates human disease. However, little is known about the gerbil gastric microbiota, and no studies have yet assessed the contribution of CagA or iron deficiency to induction of dysbiosis of the gastric mucosal microbiota. Thus, to more fully define the gerbil gastric mucosal microbiota within the context of microbial and environmental risk factors for cancer, we investigated the roles of (i) CagA in gastric dysbiosis associated with H. pylori infection, (ii) dysbiosis associated with iron deficiency within the context of H. pylori infection, and (iii) dysbiosis associated with the severity of gastric lesions along the carcinogenesis cascade.

## RESULTS

### H. pylori colonizes the gastric epithelium of Mongolian gerbils and induces inflammation and injury in a *cagA*-dependent manner.

**FIG 1:**
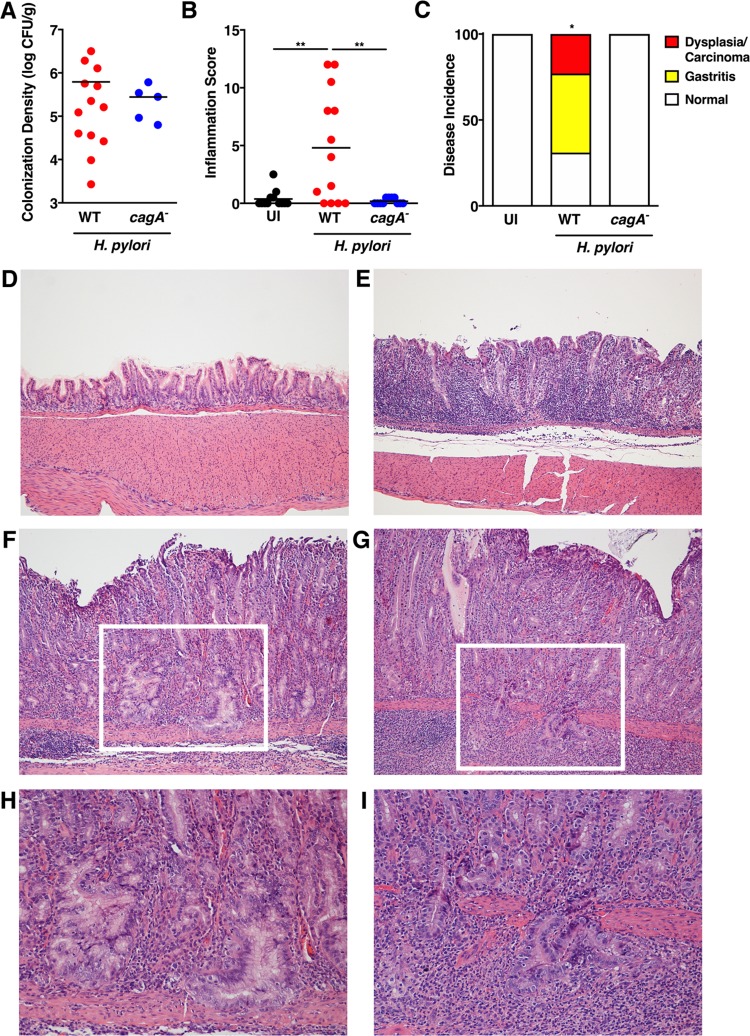
H. pylori colonizes gerbils and induces gastric inflammation and injury in a *cagA*-dependent manner. (A) Gastric tissue from uninfected gerbils (*n* = 12) or gerbils infected with wild-type (WT) H. pylori strain 7.13 (*n* = 13) or the 7.13 *cagA* isogenic mutant (*n* = 11) for 6 weeks was homogenized and plated on selective Trypticase soy agar plates with 5% sheep blood for isolation of H. pylori. Of the 11 gerbils challenged with the *cagA* isogenic mutant, CFU were detectable by quantitative culture in 5 animals. Plates were incubated for 3 to 5 days, and colonization density was determined and expressed as log CFU per gram of gastric tissue. Each data point represents colonization density from an individual animal. (B and C) Linear strips of gastric tissue, extending from the squamocolumnar junction to the proximal duodenum, were fixed in 10% neutral buffered formalin, embedded in paraffin, and stained with hematoxylin and eosin. (B and C) A pathologist, blind to the treatment groups, assessed indices of inflammation (B) and disease incidence (C). (B) The severity of acute and chronic inflammation was graded 0 to 3 (0 for no inflammation, 1 for mild, 2 for moderate, or 3 for marked inflammation) in both the gastric antrum and corpus. Each data point represents inflammation scores from an individual animal. For these analyses, both culture-positive and OTU-positive *cagA* mutant-infected gerbils were included. Values that are significantly different are indicated by bars and asterisks as follows: **, *P* < 0.001. (C) The incidence of gastric disease was also assessed, and the definitive histologic diagnosis represents the most severe lesion detected within the gastric tissue section. *, *P* = 0.005 for WT versus uninfected (UI) and *cagA* isogenic mutant. (D to G) Representative histologic images of normal gastric epithelium (D), gastritis (E), dysplasia (F), and adenocarcinoma (G) are shown at ×100 magnification (D to G). (H and I) Representative histologic images of dysplasia (H) and adenocarcinoma (I) are also shown at ×200 magnification and represent the area designated in the white boxes (F and G). Mann-Whitney U test and Fisher’s exact tests were used to determine statistical significance between uninfected and H. pylori WT- and *cagA* mutant-infected groups.

Our previous studies demonstrated that infection of Mongolian gerbils with a carcinogenic *cag^+^* H. pylori strain, strain 7.13, recapitulates key features of H. pylori-induced gastric carcinogenesis in humans (5, 17). To define the gerbil gastric mucosal microbiota under homeostatic conditions and after infection with H. pylori *in vivo*, Mongolian gerbils were challenged with sterile brucella broth as an uninfected negative control (*n* = 12), wild-type *cag^+^* carcinogenic H. pylori strain 7.13 (*n* = 13), or a 7.13 *cagA* isogenic mutant (*n* = 11). Gerbil gastric tissue was harvested 6 weeks postchallenge to assess H. pylori colonization, inflammation, and injury and composition of the gastric mucosal microbiota *in vivo*. All gerbils challenged with either the wild-type H. pylori strain 7.13 or the *cagA* isogenic mutant were successfully colonized as determined by either quantitative culture or the presence of H. pylori DNA detected by 16S rRNA sequencing. For gerbils challenged with the *cagA* isogenic mutant, in which H. pylori culture was successful (*n* = 5), colonization density levels were similar compared to animals infected with wild-type H. pylori strain 7.13 (*n* = 13) (Fig. 1A). However, some gerbils challenged with the *cagA* isogenic mutant (*n* = 6) were colonized as determined by the presence of H. pylori-specific sequences, but at levels below the limit of detection by quantitative culture. To assess the severity of inflammation, gastric tissue sections were stained with H&E and then scored for acute and chronic inflammation within the antrum and corpus of the stomach (Fig. 1B). Mongolian gerbils infected with wild-type H. pylori strain 7.13 exhibited a significant increase in gastric inflammation compared to uninfected animals (*P* < 0.005), but inflammation was attenuated following infection with the *cagA* isogenic mutant (Fig. 1B), confirming that H. pylori-induced inflammation in this model occurs in a *cagA*-dependent manner. Consistent with increased severity of inflammation, the incidence of gastric injury was detected only among gerbils infected with wild-type strain 7.13, whereby infection with this parental strain induced dysplasia and adenocarcinoma in 23% of gerbils (Fig. 1C to I), again indicating that H. pylori-induced gastric injury occurs in a *cagA*-dependent manner.

### H. pylori significantly alters the composition of the gastric mucosal microbiota in a *cagA*-dependent manner.

**FIG 2:**
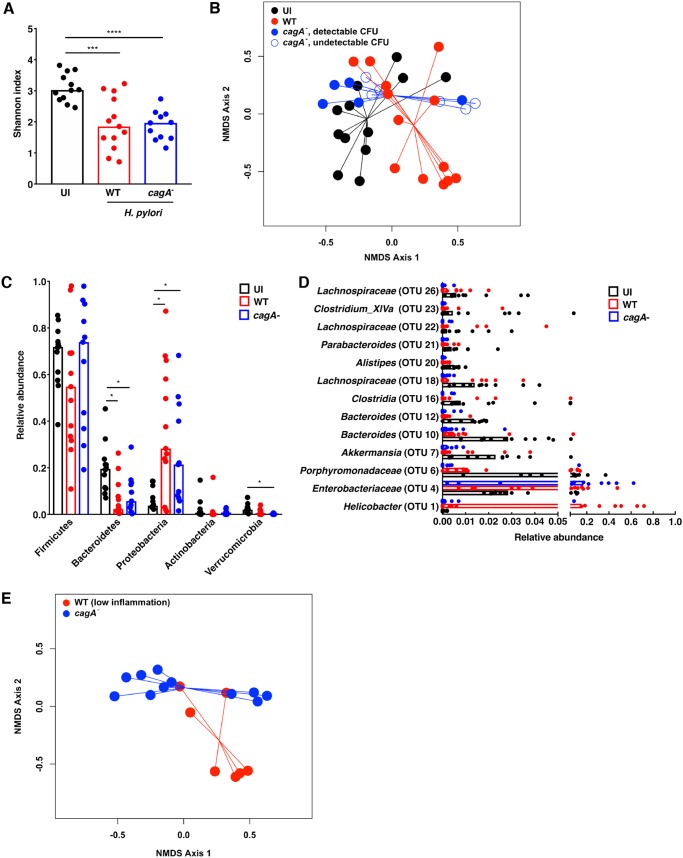
H. pylori infection alters the gastric mucosal microbiota in a *cagA*-dependent manner. Gastric tissue from uninfected (UI) gerbils (*n* = 12) or gerbils infected with wild-type (WT) H. pylori strain 7.13 (*n* = 13) or the 7.13 *cagA* isogenic mutant (*n* = 11) was harvested 6 weeks postchallenge in linear strips, extending from the squamocolumnar junction to the proximal duodenum, and then homogenized. Microbial DNA was extracted from gastric tissue and subjected to 16S rRNA gene sequencing. (A) α-Diversity of the gastric microbiota was measured by Shannon diversity metric. ***, *P* < 0.0005; ****, *P* < 0.0001. (B) β-Diversity of the gastric microbiota was measured by Yue and Clayton’s measure of dissimilarity and is shown in a nonmetric multidimensional scaling (NMDS) plot. Blue closed circles indicate *cagA* isogenic mutant with detectable CFU (*n* = 5), while blue open circles indicate *cagA* isogenic mutant without detectable CFU (*n* = 6). Uninfected versus infected with strain 7.13, *P* = 0.001; uninfected versus infected with *cagA* mutant, *P* < 0.001; infected with strain 7.13 versus infected with *cagA* mutant, *P* = 0.006. (C) The relative abundances of phyla within the gastric microbiota were determined. *, *P* < 0.05. (D) Operational taxonomic units (OTUs) were measured by the linear discriminant analysis (LDA) effect size (LEfSe) algorithm and are shown as scatter plots. Statistical significance is indicated in the text. (E) β-Diversity of the gastric microbiota was measured by Yue and Clayton’s measure of dissimilarity and is shown in a nonmetric multidimensional scaling plot in samples stratified by the severity of gastric inflammation. Infected with strain 7.13 with low inflammation (*n* = *7)* versus infected with *cagA* mutant (*n* = 11), *P* = 0.041.

To next define potential dysbiosis induced by H. pylori infection and specifically the oncoprotein CagA *in vivo*, gastric tissue from uninfected gerbils or gerbils infected with wild-type H. pylori *cag^+^* strain 7.13 or the *cagA* isogenic mutant was harvested in linear strips, extending from the squamocolumnar junction to the proximal duodenum, and then homogenized to facilitate assessment of the mucosal microbiota throughout the stomach. Microbial DNA was extracted from gastric tissue and subjected to 16S rRNA gene sequencing for gastric mucosal microbiota analyses. For these studies, all samples from gerbils infected with the *cagA* isogenic mutant (*n* = 11) were included. α-Diversity (relative species diversity) was first measured using the Shannon diversity metric (Fig. 2A). Infection with either wild-type H. pylori strain 7.13 (*n* = 13; *P* = 0.0004) or the *cagA* mutant (*n* = 11; *P* < 0.0001) significantly decreased α-diversity within the stomach compared to uninfected controls. β-Diversity was next measured using Yue and Clayton’s measure of dissimilarity, which quantifies the ratio between α-diversity and regional diversity, yielding a more accurate assessment of microbial community structure. These findings revealed that the structure of the mucosal microbial community of uninfected gerbils (*n* = 12) is significantly different from gerbils infected with either wild-type H. pylori strain 7.13 (*n* = 13; *P* = 0.001) or the *cagA* isogenic mutant (*n* = 11; *P* < 0.001) (Fig. 2B). Further, microbial community structure of gerbils infected with wild-type H. pylori strain 7.13 was significantly different than the structure in gerbils infected with the *cagA* isogenic mutant (*P* = 0.006) (Fig. 2B), indicating that infection with H. pylori leads to alterations in the structure of the microbial community in a *cagA*-dependent manner. Importantly, despite differences in colonization burden among gerbils infected with the *cagA* isogenic mutant, significant differences in microbial community structure persisted regardless of colonization density levels, such that the microbial community of uninfected gerbils was significantly different from gerbils infected with the *cagA* isogenic mutant with detectable CFU (*n* = 5; *P* = 0.012) or without detectable CFU (*n* = 6; *P* = 0.001) (Fig. 2B). In addition, there were no significant differences in microbial community structure among gerbils infected with the *cagA* isogenic mutant with (*n* = 5) or without detectable CFU (*n* = 6) (*P* = 0.166) (Fig. 2B). Finally, the microbial community of gerbils infected with wild-type strain 7.13 (*n* = 13) was also significantly different from gerbils infected with the *cagA* isogenic mutant with (*n* = 5; *P* = 0.029) or without detectable CFU (*n* = 6; *P* = 0.031) (Fig. 2B). These data suggest that infection with the *cagA* isogenic mutant alters microbial community structure differently than wild-type H. pylori strain 7.13 in a manner that is not dependent upon colonization burden.

To assess changes in the structure of the gastric microbial communities in greater depth, the relative abundance of phyla (Fig. 2C) and operational taxonomic units (OTUs) (Fig. 2D) were next ascertained. As expected, infection with either H. pylori strain resulted in a significant enrichment of *Proteobacteria* (*P* < 0.001), likely driven by H. pylori colonization, and concomitant significant decreases in *Bacteroidetes* (*P* < 0.01) compared to uninfected controls (Fig. 2C). We next assessed the relative abundance of OTUs among H. pylori-infected gerbils compared to uninfected gerbils and, as expected, found significant increases in *Helicobacter* OTUs (*P* = 0.001) (Fig. 2D). The lower levels of *Helicobacter* OTUs among gerbils infected with the *cagA* isogenic mutant are likely due to lower colonization burdens observed among these animals. In addition to *Helicobacter*, a significant increase in Enterobacteriaceae (*P* = 0.02) was also observed among H. pylori-infected gerbils (Fig. 2D). Decreases in specific OTUs were also observed following H. pylori infection, which included significant reductions in the relative abundance of *Porphyromonadaceae* (*P* < 0.04), *Akkermansia* (*P* < 0.006), *Bacteroides* (*P* < 0.003), and *Lachnospiraceae* (*P* < 0.02) (Fig. 2D). We next focused on the relative abundance of OTUs among gerbils infected with wild-type H. pylori 7.13 (*n* = 13) compared to gerbils infected with the *cagA* isogenic mutant (*n* = 11). Consistent with significant alterations in microbial community structure that occurred in a *cagA*-dependent manner (Fig. 2B), gerbils infected with the *cagA* isogenic mutant exhibit significant decreases in *Akkermansia* (*P* = 0.005), *Bacteroides* (*P* < 0.04), and *Lachnospiraceae* (*P* = 0.03) compared to gerbils infected with the wild-type strain, again indicating that CagA exerts an important role in sculpting the composition of the H. pylori-colonized gastric microbiota (Fig. 2D).

Finally, since we demonstrated that infection with wild-type H. pylori strain 7.13 induced significantly higher levels of inflammation and injury compared to infection with the *cagA* isogenic mutant (Fig. 1B to I), we next compared the gastric microbiota of gerbils infected with wild-type H. pylori strain 7.13 (*n* = 7) that harbored relatively low levels of gastric inflammation (inflammation scores of 0 or 1) to gerbils infected with the *cagA* isogenic mutant (*n* = 11) to determine the potential role of inflammation in *cagA*-dependent gastric dysbiosis (Fig. 2E). β-Diversity among gerbils infected with the *cagA* isogenic mutant (*n* = 11) remained significantly different from either gerbils infected with wild-type H. pylori strain 7.13 that developed only minimal inflammation (*n* = 7; *P* = 0.041) (Fig. 2E) or gerbils infected with wild-type H. pylori strain 7.13 that developed high levels of inflammation (*n* = 6; *P* = 0.011; data not shown), suggesting that *cagA*-dependent gastric dysbiosis is not driven solely by the severity of gastric inflammation induced by H. pylori. Finally, there were no significant differences in β-diversity among gerbils infected with wild-type H. pylori strain 7.13 that developed low levels (*n* = 7) versus high levels (*n* = 6) of gastric inflammation (*P* = 0.567; data not shown).

### Host iron levels fail to alter the diversity or composition of the gastric mucosal microbiota.

**FIG 3:**
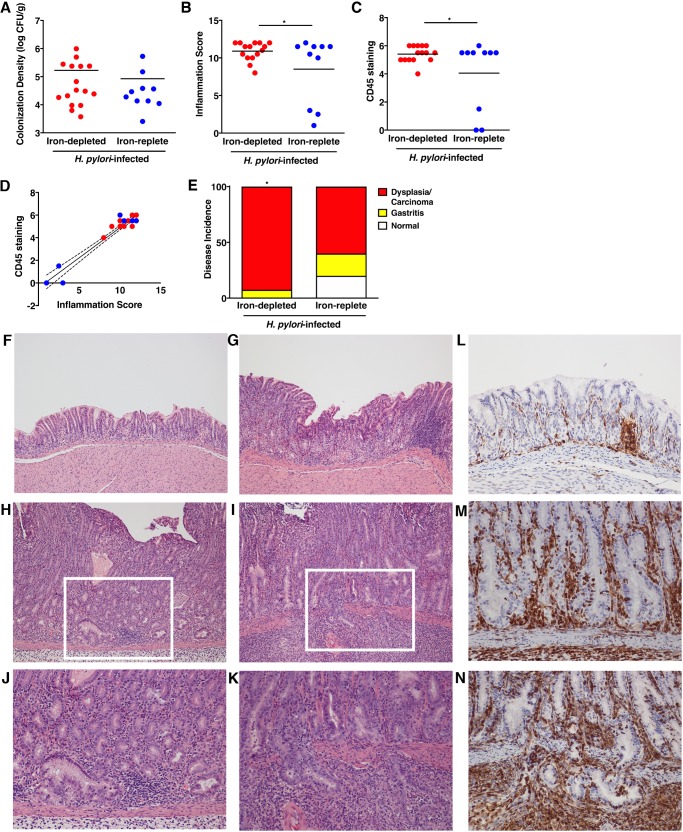
H. pylori colonizes gerbils independent of host iron status and induces inflammation and injury in an iron-dependent manner. (A) Gastric tissue from uninfected gerbils or gerbils infected with wild-type H. pylori strain 7.13 maintained on either iron-depleted (*n* = 16) or iron-replete (*n* = 10) diets was harvested 6 weeks postchallenge, homogenized, and plated on selective Trypticase soy agar plates with 5% sheep blood for isolation of H. pylori. The plates were incubated for 3 to 5 days, and colonization density was determined and expressed as log CFU per gram of gastric tissue. Each data point represents colonization density from an individual animal. (B to N) Linear strips of gastric tissue, extending from the squamocolumnar junction to the proximal duodenum, were fixed in 10% neutral buffered formalin, embedded in paraffin, and stained with hematoxylin and eosin or an antibody targeting CD45. A pathologist, blind to the treatment groups, assessed indices of inflammation (B), levels of CD45 staining (C), correlations between CD45 staining and inflammation (*P* < 0.0001) (D), and disease incidence (E). (B) Severity of acute and chronic inflammation was graded 0 to 3 (0 for no inflammation, 1 for mild, 2 for moderate, or 3 for marked inflammation) in both the gastric antrum and corpus. Each data point represents inflammation scores from an individual animal. *, *P* < 0.05. Animals challenged with brucella broth are not shown, but they exhibited no evidence of H. pylori colonization, inflammation, or histologic injury. (C) The extent of CD45 staining was graded 0 to 3 (0 for minimal staining, 1 for mild, 2 for moderate, or 3 for marked) in both the gastric antrum and corpus. Each data point represents scores from an individual animal. (D) The correlation between CD45 staining and gastric inflammation was determined (*P* < 0.0001). (E) The incidence of gastric disease was also assessed, and the definitive histologic diagnosis represents the most severe lesion detected within the gastric tissue section. *, *P* = 0.05, iron-depleted versus iron-replete. (F to I) Representative histologic images of normal gastric epithelium (F), gastritis (G), dysplasia (H), and adenocarcinoma (I) are shown at ×100 magnification. (J and K) Representative histologic images of dysplasia (J) and adenocarcinoma (K) are also shown at ×200 magnification and represent the area designated in the white boxes in panels H and I. (L to N) Representative immunohistochemistry images of CD45 staining in gastritis (L), dysplasia (M), and adenocarcinoma (N) at ×200 magnification are shown. Mann-Whitney U test and Fisher’s exact and linear regression tests were used to determine statistical significance between H. pylori-infected groups.

Our previous studies demonstrated that use of an iron-depleted diet significantly augments H. pylori-induced gastric inflammation and accelerates carcinogenesis in gerbils (5). Thus, to define changes in the gerbil gastric microbiota and dysbiosis associated with iron deficiency in conjunction with H. pylori infection, we maintained gerbils on iron-depleted or iron-replete diets and then challenged gerbils with sterile brucella broth or wild-type *cag^+^* carcinogenic H. pylori strain 7.13. Gerbil gastric tissue was harvested 6 weeks postchallenge to assess H. pylori colonization, inflammation, and changes in composition of the gastric microbiota *in vivo*. All challenged gerbils were successfully colonized, and colonization density was similar among infected gerbils maintained on iron-depleted (*n* = 16) or iron-replete (*n* = 10) diets (Fig. 3A). Gerbils infected with wild-type H. pylori strain 7.13 exhibited a significant increase in gastric inflammation regardless of diet, but as expected, inflammation was significantly elevated among H. pylori-infected gerbils maintained on an iron-depleted diet (*P* = 0.05) (Fig. 3B). To independently assess gastric inflammation within gerbil gastric mucosa in a quantitative manner, immunohistochemistry was performed on gerbil gastric tissue sections. CD45 leukocyte common antigen was used as a marker of CD45^+^ myeloid and lymphoid cell populations. The levels of CD45 were significantly elevated among infected gerbils maintained on either iron-depleted or iron-replete diets compared to uninfected gerbils but were significantly higher among infected gerbils maintained on iron-depleted diets (*P* = 0.05) and, importantly, directly correlated with levels of gastric inflammation (*P* < 0.0001) (Fig. 3C, D, and L to N). Consistent with the increased severity of gastric inflammation, the incidence of gastric injury was augmented among H. pylori-infected gerbils maintained on iron-depleted diets (*P* = 0.05) (Fig. 3E to K). Specifically, the incidence of gastric dysplasia and adenocarcinoma increased from 60% among H. pylori-infected gerbils maintained on iron-replete diets to 93% among gerbils maintained on iron-depleted diets (Fig. 3E to K).

**FIG 4:**
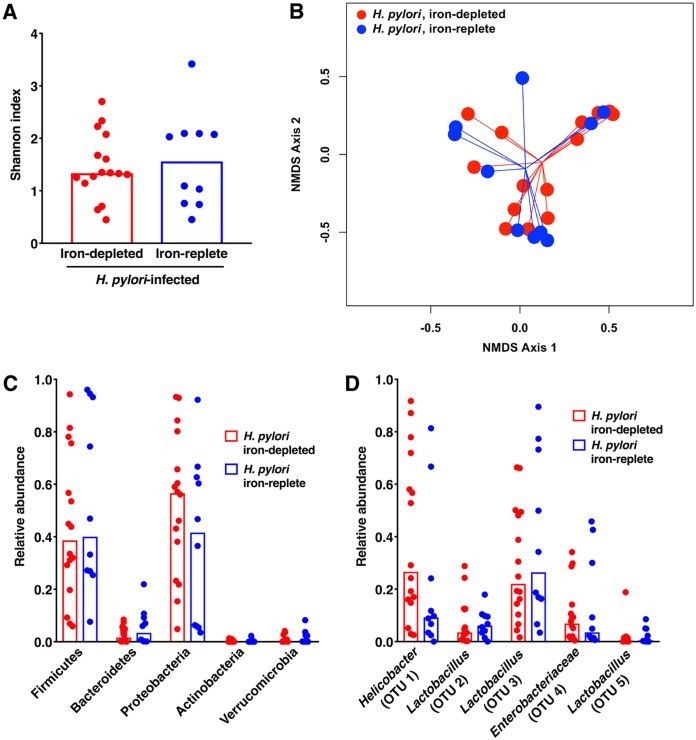
Host iron levels fail to alter the diversity or composition of the gastric mucosal microbiota. Gastric tissue from uninfected gerbils or gerbils infected with wild-type H. pylori strain 7.13 maintained on either iron-depleted (*n* = 16) or iron-replete (*n* = 10) diets was harvested 6 weeks postchallenge in linear strips, extending from the squamocolumnar junction to the proximal duodenum, and then homogenized. Microbial DNA was extracted from gastric tissue and subjected to 16S rRNA gene sequencing. (A) α-Diversity of the gastric microbiota was measured by Shannon diversity metric among H. pylori-infected gerbils maintained on either iron-depleted or iron-replete diets. (B) β-Diversity of the gastric microbiota was measured by Yue and Clayton’s measure of dissimilarity and is shown in a nonmetric multidimensional scaling plot among H. pylori-infected gerbils maintained on either iron-depleted or iron-replete diets. (C) The relative abundance of phyla within in the gastric microbiota was determined among H. pylori-infected gerbils maintained on either iron-depleted or iron-replete diets. (D) Operational taxonomic units (OTUs) were measured by the LDA effect size (LEfSe) algorithm among H. pylori-infected gerbils maintained on either iron-depleted or iron-replete diets.

Despite differences in gastric inflammation and injury, there were no significant differences in α-diversity or β-diversity of the gastric microbiota between H. pylori-infected gerbils maintained on iron-depleted (*n* = 16) versus iron-replete (*n* = 10) diets (Fig. 4A and B). There were also no significant differences in the α- or β-diversity of the gastric microbiota among uninfected gerbils maintained on either iron-replete or iron-depleted diets (data not shown). Similarly, when the relative abundances of phyla (Fig. 4C) and OTUs (Fig. 4D) were assessed, there were no significant differences observed between H. pylori-infected gerbils maintained on iron-depleted versus iron-replete diets. Consistent with the previous data (Fig. 2), the gastric microbiota of H. pylori-infected gerbils maintained on either iron-depleted or iron-replete diets was dominated by *Proteobacteria* (Fig. 4C), which corresponded to enrichment of *Helicobacter* and Enterobacteriaceae OTUs (Fig. 4D) and *Firmicutes* (Fig. 4C), which corresponded to a predominance of *Lactobacillus* OTUs (Fig. 4D).

### The degree of H. pylori-induced gastric injury significantly alters diversity and community structure of the gastric mucosal microbiota, independent of host iron status.

**FIG 5:**
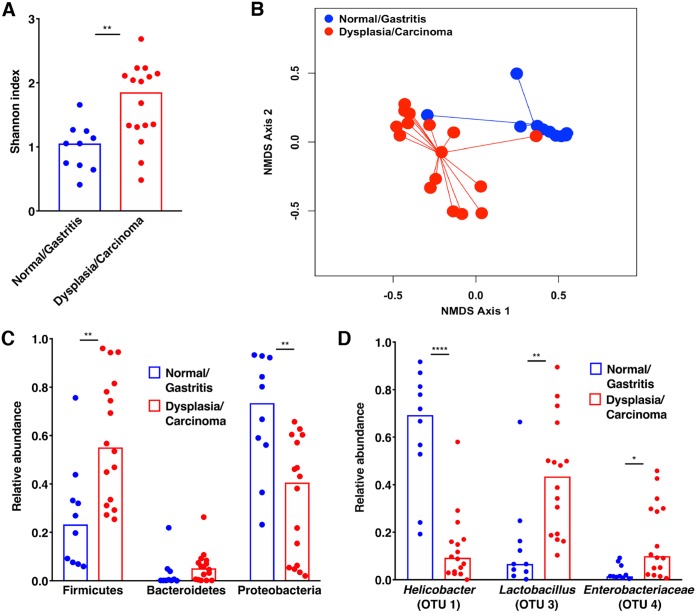
The severity of histologic gastric injury following H. pylori infection significantly alters the diversity and structure of gastric mucosal microbiota, independent of host iron status. Gastric tissue from uninfected gerbils or gerbils infected with wild-type H. pylori strain 7.13 maintained on either iron-depleted or iron-replete diets was harvested 6 weeks postchallenge in linear strips, extending from the squamocolumnar junction to the proximal duodenum, and then homogenized. Microbial DNA was extracted from gastric tissue and subjected to 16S rRNA gene sequencing. (A) α-Diversity of the gastric microbiota was measured by Shannon diversity metric among H. pylori-infected gerbils maintained on either iron-depleted or iron-replete diets and stratified based on the severity of histologic injury. (B) β-Diversity of the gastric microbiota was measured by Yue and Clayton’s measure of dissimilarity and is shown in a nonmetric multidimensional scaling plot among H. pylori-infected gerbils maintained on either iron-depleted or iron-replete diets and stratified based on the severity of histologic injury. Normal/Gastritis (*n* = 10) versus Dysplasia/Carcinoma (*n* = 16), *P* < 0.001. (C) The relative abundance of phyla within the gastric microbiota was determined among H. pylori-infected gerbils maintained on either iron-depleted or iron-replete diets and stratified based on the severity of histologic injury. (D) The LDA effect size (LEfSe) algorithm was used to identify operational taxonomic units (OTUs) that were differentially abundant among H. pylori-infected gerbils maintained on either iron-depleted or iron-replete diets and stratified based on the severity of histologic injury. *, *P*, <0.01; **, *P* < 0.001; ****, *P* < 0.00001.

Having observed no differences in microbial structure or composition of the gastric mucosal microbiota among H. pylori-infected gerbils maintained on iron-depleted versus iron-replete diets, we next postulated that dysbiosis in the gastric microbiota may instead be associated with the severity of injury that develops along the gastric carcinogenesis cascade during a 6-week infection. Samples were stratified based on histologic diagnosis, which ranged from normal to gastritis, dysplasia, and adenocarcinoma. Independent of iron status, α-diversity of the gastric microbiota significantly differed among H. pylori-infected gerbils harboring premalignant and malignant lesions (*n* = 16; *P* = 0.001) compared to H. pylori-infected gerbils with normal histology or gastritis alone (*n* = 10; Fig. 5A). β-Diversity also significantly differed among gerbils with increasing severity of histologic injury, whereby gerbils with normal gastric mucosa or gastritis alone (*n* = 10) significantly differed from gerbils with gastric dysplasia or adenocarcinoma (*n* = 16; *P* < 0.001) (Fig. 5B).

To more directly assess changes in the structure of the gastric microbial communities within the context of histologic injury, the relative abundance of phyla (Fig. 5C) and OTUs (Fig. 5D) were quantified. The relative abundance of *Proteobacteria* was significantly decreased in samples with gastric dysplasia or adenocarcinoma compared to samples with normal histology or gastritis alone (*P* = 0.001) (Fig. 5C), which was likely attributable to decreased abundance of *Helicobacter* OTUs (*P* < 0.00001) (Fig. 5D). As a result, the relative abundance of *Firmicutes* was significantly increased in samples with gastric dysplasia or adenocarcinoma compared to cases with normal histology or gastritis alone (*P* < 0.005) (Fig. 5C), which was attributable to increased abundance of *Lactobacillus* OTUs (*P* < 0.01) and Enterobacteriaceae OTUs (*P* = 0.01) (Fig. 5D). Collectively, these data demonstrate that H. pylori infection significantly decreases the diversity of the gastric mucosal microbiota in a *cagA-*dependent manner, and further, that as carcinogenesis progresses, there are corresponding alterations in microbial diversity and the structure of the microbial community within the stomach that parallel the severity of disease.

## DISCUSSION

The human gastric microbiota is highly diverse, and colonization with H. pylori has been shown to modify this diversity (18–20). However, while studies have established that the gastric microbiota is significantly altered among patients during progression to gastric cancer, this has been reported to occur in different patterns (21–26). For example, Dicksved et al. observed no significant differences in the complexity of the gastric microbiota between patients with dyspepsia or gastric cancer (21), while Eun et al. demonstrated an increase in the diversity of the gastric microbiota in gastric cancer specimens compared to specimens with chronic gastritis (23). However, several other studies have demonstrated reduced microbial diversity in biopsy specimens from patients with gastric cancer compared to biopsy specimens from patients with chronic gastritis alone (22, 25, 26). A common theme for most of these studies is that the abundance of *Helicobacter* decreases as disease worsens (21–26), potentially allowing for greater microbial diversity.

Consistent with these studies, we also demonstrated a significant decrease in the presence of *Helicobacter* species with increasing severity of disease. In addition, we found increased microbial diversity among samples with gastric adenocarcinoma and consistent with Dicksved et al. (21) and Aviles-Jimenez et al. (22), we also found significant increases in *Lactobacillus* species among gastric tissue samples with adenocarcinoma. There are potential explanations for such different results. One explanation is that inherent differences exist between human populations and animal models. In addition, many previous studies specifically evaluated microbial populations from biopsy specimens targeting specific loci of gastric carcinoma or other disease states. The current work analyzed gastric mucosal microbiota from linear strips of the stomach, extending from the squamocolumnar junction to the proximal duodenum, which provides a much broader global assessment of the gastric microbial community. In addition to differences in populations and sampling of gastric tissue, the increase in microbial diversity observed in advanced stages of disease may also be due to atrophy that occurs during disease progression, which ultimately results in the loss of parietal cells and reductions in acid secretion (27). Increases in gastric pH provide a more hospitable gastric niche to sustain growth of diverse microbial species, which may contribute to the increase in diversity and community structure of the gastric microbiota.

Currently, little is known about the composition of the gerbil gastric microbiota; however, Sun et al. assessed alterations in the gerbil gastric microbiota before and after H. pylori infection and demonstrated that *Lactobacillus* was the dominant component of both uninfected and H. pylori-infected gerbil stomachs (28), which was also confirmed by Zaman et al. (29). In another study of uninfected and H. pylori-infected gerbils, Osaki et al. also found an abundance of *Lactobacillus*, but also *Bifidobacterium*, *Clostridia*, and *Enterococcus* among both H. pylori-infected and uninfected gerbils; however, the abundance of *Bifidobacterium* and *Clostridia* were significantly lower among H. pylori-negative gerbils (30). Similar to these studies, we also observed an abundance of *Lactobacillus*, but other abundant OTUs were different and included Enterobacteriaceae and *Porphyromonadaceae*, among others.

We also sought to define the gerbil gastric microbiota within the context of both microbial and environmental risk factors for gastric cancer. Importantly, we found that that the H. pylori virulence factor CagA significantly contributes to gastric dysbiosis, while iron deficiency did not. Interestingly, the *cagA*-dependent alterations in the gastric microbiota occurred independent of the inflammatory response, suggesting that CagA *per se* directly affects microbial community structure. Ohnishi et al. demonstrated that transgenic mice overexpressing CagA alone develop significant gastric epithelial cell hyperproliferation and gastric adenocarcinoma (2); our studies have now demonstrated that in addition to the role of CagA facilitating H. pylori-induced injury, it also plays a major role in H. pylori-induced gastric dysbiosis. CagA plays a pivotal role in altering host cell signaling cascades within gastric epithelial cells, which may also sculpt the gastric microbiota. For example, CagA has been shown to activate NF-κB, which mediates downstream proinflammatory signaling responses important for inducing inflammation (31); however, H. pylori has been shown to induce beta-defensins in gastric epithelial cells in a *cagA*- and NF-κB-dependent manner (32–34). Defensin production may therefore contribute to the composition of the microbiota. Importantly, H. pylori has also been shown to induce reactive oxygen species and oxidative stress in a *cagA*-dependent manner (35, 36). We therefore speculate that aberrant inflammatory and potentially antimicrobial environments that arise within the context of infection by *cagA^+^* strains of H. pylori likely shape the diversity and community structure of gastric microbiota in a CagA-dependent manner. In addition to altering the inflammatory milieu, H. pylori has been shown to directly inhibit acid secretion by parietal cells in a T4SS-, CagA-, and NF-κB-dependent manner *in vitro* (37–39). *In vivo*, H. pylori infection also leads to the loss of parietal cells and increases in gastric pH within the Mongolian gerbil model (5, 40). It is therefore likely that increases in gastric pH engender a less hostile environment that facilitates the diversification of microbial species in this niche, which may contribute to the increased diversity and alterations in community structure of the gastric microbiota that occur in a CagA-dependent manner in this model.

There have been other *in vivo* models used to investigate H. pylori and the gastric microbiota. Inbred mice with defined genotypes are one commonly used model, and the ability of H. pylori to alter the gastric microbiota of mice is mediated by several factors, including the murine genetic background and source of the animals. Rolig et al. determined, using uninfected and infected C57BL/6 mice, that *Firmicutes*, *Bacteroidetes*, *Proteobacteria*, and *Actinobacteria*, were the most predominant phyla, with *Firmicutes* accounting for greater than 50% of the isolates (41), similar to what has been reported in the human stomach. In H. pylori-infected mice, the abundance of *Firmicutes*, *Bacteroidetes*, and *Proteobacteria* decreased, while certain subpopulations, including *Helicobacter*, *Clostridia*, and *Verrucomicrobia* increased (41). Interestingly, this study also demonstrated that despite equal levels of colonization with H. pylori, C57BL/6 mice from two independent vendors developed different intensities of H. pylori-induced inflammation and that these differences were mediated by the presence of different ratios of *Lactobacillus* species in the gastric microbiota (41). Thus, despite mice having identical genetic backgrounds, the commercial source can dramatically affect the composition of the gastric microbiota.

In terms of disease, recent studies of mice have provided evidence of a potential role of the non-H. pylori microbiota in H. pylori-induced gastric carcinogenesis. Specifically, the gastric microbiota was shown to accelerate and enhance the development of preneoplastic lesions and adenocarcinoma in transgenic INS-GAS mice, which are genetically predisposed to gastric cancer. Lofgren et al. demonstrated that INS-GAS mice harboring a complex microbiota spontaneously developed gastric cancer within 7 months, whereas the development of gastric cancer was markedly prolonged in germfree INS-GAS mice (42). Consistent with other studies, Lofgren et al. also observed a significant decrease in the overall diversity of microbiota following infection with H. pylori (42). These observations were studied in greater depth in an INS-GAS mono-associated H. pylori model, where the addition of only three species of commensal bacteria (ASF356 *Clostridium* species, ASF361 Lactobacillus murinus, and ASF519 *Bacteroides* species) was sufficient to promote gastric neoplasia in H. pylori-infected INS-GAS mice to the same extent as observed in H. pylori-infected INS-GAS mice harboring a complex microbiota (43). Further supporting the importance of the gastric microbiota in disease, interventions with antibiotic therapy delayed the onset of gastric cancer in INS-GAS mice, regardless of the presence of H. pylori (44). Collectively, these results suggest that the H. pylori can act synergistically with a restricted gastric microbiota to promote gastric neoplasia.

In conclusion, despite differences among the various models and studies, the evidence that the microbiota is essential to promote health and prevent disease (6) and that dysbiosis contributes to host susceptibility to infection and disease (7) is strong. Although great advances have been made in understanding complex interactions between the gastric microbiota and H. pylori in the development of gastric inflammation and cancer, studies of these microbial populations in humanized animal models in the future may yield strategies for manipulating the gastric microbiota to prevent the development of gastric cancer.

## MATERIALS AND METHODS

### Helicobacter pylori culture.

The wild-type carcinogenic *cagA^+^* H. pylori strain 7.13 and its *cagA* isogenic mutant were minimally passaged on Trypticase soy agar plates with 5% sheep blood (BD Biosciences) and then grown in brucella broth (BD Biosciences) supplemented with 10% fetal bovine serum (FBS) (Atlanta Biologicals) for 16 h at 37°C with 5% CO_2_. The H. pylori 7.13 *cagA* isogenic mutant was grown on brucella broth agar (BD Biosciences) plates supplemented with kanamycin (Sigma-Aldrich) (20 μg/ml) for selection prior to overnight cultures.

### Mongolian gerbil model.

Outbred male Mongolian gerbils were purchased from Charles River Laboratories and cohoused in the Vanderbilt University Animal Care Facilities until infection with H. pylori to control for cage effects. Gerbils were maintained on the standard rodent diet or modified diets (TestDiet AIN-93M [Purina Feed, LLC] that contained 0 ppm iron [iron-depleted; TestDiet 5TWD] or 250 ppm iron [iron-replete; TestDiet 5STQ]), as previously described (5). Gerbils were orogastrically challenged with sterile brucella broth as a negative control, wild-type *cag^+^* H. pylori strain 7.13, or a 7.13 *cagA* isogenic mutant, and all gerbils were euthanized 6 weeks after challenge, as previously described (5). The Vanderbilt University Institutional Animal Care and Use Committee (IACUC) approved all experiments and procedures.

### H. pylori quantitative culture.

Linear strips of gastric tissue, extending from the squamocolumnar junction through the proximal duodenum, were harvested 6 weeks postchallenge and homogenized in sterile phosphate-buffered saline (PBS) (Corning). Following serial dilution, samples were plated on selective Trypticase soy agar (TSA) (Remel) plates with 5% sheep blood (Hemostat Lab) containing vancomycin (Sigma-Aldrich) (20 μg/ml), nalidixic acid (Sigma-Aldrich) (10 μg/ml), bacitracin (Calbiochem) (30 μg/ml), and amphotericin B (Sigma-Aldrich) (2 μg/ml) for selection, isolation, and quantification of H. pylori. The plates were incubated for 3 to 5 days at 37°C with 5% CO_2_. Colony counts were expressed as log CFU per gram of gastric tissue. For gerbils in which CFU was below the limit of detection, H. pylori colonization was determined by the presence of H. pylori DNA detected by 16S rRNA sequencing.

### Histopathology.

Linear strips of gastric tissue, extending from the squamocolumnar junction to the proximal duodenum, were harvested 6 weeks postchallenge and fixed in 10% neutral buffered formalin (Azer Scientific), embedded in paraffin, and stained with hematoxylin and eosin (H&E). A single pathologist (M. B. Piazuelo), blind to treatment groups, assessed and scored indices of inflammation and injury 6 weeks postchallenge. The severity of acute and chronic inflammation was graded on a scale from 0 to 3 (inflammation scored as follows: 0 for no inflammation, 1 for mild, 2 for moderate, or 3 for marked inflammation) in both the gastric antrum and corpus, leading to a maximum cumulative score of 12. The presence of gastric dysplasia and adenocarcinoma were also assessed, and the definitive histologic diagnosis represents the most severe lesion detected within the gastric tissue section.

### Immunohistochemistry.

Linear strips of gastric tissue, extending from the squamocolumnar junction to the proximal duodenum, were harvested 6 weeks postchallenge and fixed in 10% neutral buffered formalin (Azer Scientific), embedded in paraffin, and stained with an antibody targeting CD45 (leukocyte common antigen; Abcam) for detection of CD45^+^ myeloid and lymphoid cell populations. A single pathologist (M. B. Piazuelo), blind to treatment groups, assessed and scored CD45 staining on a scale from 0 to 3 (staining scored as follows: 0 for minimal staining, 1 for mild, 2 for moderate, or 3 for marked) in both the gastric antrum and corpus for a total maximal score of 6.

### Microbial DNA extraction and 16S rRNA gene sequencing.

Linear strips of gastric tissue, extending from the squamocolumnar junction to the proximal duodenum were harvested 6 weeks postchallenge and immediately frozen at −80°C. For analysis of the microbiota, gastric tissue samples were homogenized in PowerMag bead solution (MO BIO Laboratories) with 50 mM TCEP solution (Thermo Scientific). Microbial genomic DNA was extracted using the PowerSoil DNA isolation kit (MO BIO Laboratories), according to the manufacturer’s instructions. Sterile water mock extractions were processed in parallel to gastric tissues to control for reagent contamination. The V4 region of the 16S rRNA gene from each sample was amplified and sequenced using the Illumina Sequencing platform within the Vanderbilt Technologies for Advanced Genomics Laboratory. Sequences were curated using mothur software package (45), as previously described and performed (46). Sequences were aligned to the SILVA 16S rRNA sequence database (47), and chimeric sequences were identified by UCHIME (48) and removed. After curation, between 72 and 242,627 sequences (median, 9,774.5) with a median length of 253 bp were obtained. To minimize the impact of uneven sampling on downstream analyses, the number of sequences in each sample was rarefied to 1,000.

### Gastric microbiota analyses.

Sequences were clustered into OTUs based on a 3% distance cutoff calculated using the OptiClust algorithm. All sequences were classified using the RDP training set (version 16), and OTUs were assigned a classification based on the taxonomy that had the majority consensus of sequences within each OTU using a naive Bayesian classifier (49). α-Diversity was calculated using the Shannon diversity index, while β-diversity was calculated using the θ_YC_ distance metric with OTU frequency data (50).

### Statistical analyses.

Statistical analyses were performed using GraphPad Prism and R. The Whitney U test and ANOVA test were used for comparison of H. pylori colonization and inflammation and Fisher’s exact tests were used for disease incidence. Linear regression was used to determine correlation between CD45 staining and inflammation. Welch’s *t* test was used for comparisons of continuous variables between two treatment groups. Analysis of molecular variance (AMOVA) was performed to determine significance between the community structures of different groups of samples based on θ_YC_ distance matrices (51). The biomarker discovery algorithm LEfSe (linear discriminant analysis [LDA] effect size) was implemented to identify features (OTUs) differentially abundant in each group and to further assess gastric microbiota variation between dietary groups (52). Negative mock extractions were processed and analyzed together with all study samples, but we did not observe significant overlap with gastric tissue samples based on β-diversity metrics.

### Data availability.

FASTQ sequence data used in this study have been deposited to the Sequence Read Archive (SRA) at NCBI under the BioProject accession number PRJNA521338.
